# Pharmacological Properties and Phytochemical Profile of *Sargassum filipendula* Extracts

**DOI:** 10.3390/md24050153

**Published:** 2026-04-26

**Authors:** Varun Jaiswal, Hae-Jeung Lee

**Affiliations:** 1 Department of Food and Nutrition, College of BioNano Technology, Gachon University, 1342 Seongnam-daero, Sujeong-gu, Seongnam-si 13120, Republic of Korea; computationalvarun@gmail.com; 2 Institute for Aging and Clinical Nutrition Research, Gachon University, Seongnam-si 13120, Republic of Korea; 3 Department of Health Sciences and Technology, Gachon Advanced Institute for Health Sciences and Technology (GAIHST), Gachon University, Incheon 21999, Republic of Korea

**Keywords:** *Sargassum filipendula*, phytochemicals, pharmacological activities, anticancer, fucoidan, phlorotannins, immunomodulation, antioxidant

## Abstract

*Sargassum filipendula* is a widely distributed, edible brown alga that possesses a rich nutritional profile. Several studies have demonstrated that the components/extracts of *S. filipendula* (SFE) possess diverse pharmacological potential against both infectious and non-infectious diseases. These include antibacterial and antifungal properties, as well as antioxidant, anti-aging, anti-osteoporosis, antiviral, antiprotozoal, and immunomodulatory effects. Furthermore, SFE has shown significant anticancer activity across various malignant cell lines. The unique phytochemical profile of this species, characterized by the presence of sulfated polysaccharides (primarily fucoidan), carotenoids, phenols, glycolipids, and phlorotannins, serves as the foundation for these wide-ranging pharmacological activities. Studies have demonstrated that SFE can modulate key molecular targets, such as glycogen synthase kinase-3 beta, and activate the mitochondrial-dependent apoptosis pathway, providing a robust mechanistic basis for the observed pharmacological activities. Recent evaluations of nutritional parameters and techno-functional properties confirm the rich nutritional profile of *S. filipendula*, supporting its application in a diverse range of food products. Despite its diverse bioactive phytochemicals and broad efficacy against infectious and non-infectious diseases, research on *S. filipendula* remains largely restricted to in vitro preclinical studies. The lack of a comprehensive compilation of its pharmacological activities, phytochemical profiles, and molecular targets hinders its development as a therapeutic agent. This review aims to bridge this gap by compiling the existing knowledge, identifying research deficiencies, particularly the lack of in vivo data and safety assessments for high-dose therapeutic applications, while proposing suggestions for transitioning *S. filipendula* into a viable therapeutic or functional supplement.

## 1. Introduction

Seaweeds, or macroalgae, are rich in bioactive compounds, such as sulfated polysaccharides, polyphenols, and carotenoids, which can exert beneficial effects on health and may reduce the risk of chronic diseases when consumed as part of a balanced diet [1,2,3]. Consequently, marine macroalgae offer significant potential as functional foods that could mitigate disease onset, particularly in populations affected by poor dietary habits and nutritional deficiencies, as they provide an affordable and accessible nutrient source [2,4]. Beyond general wellness, seaweeds have garnered substantial scientific attention as potent natural sources of therapeutic agents against critical illnesses, most notably in the fields of oncology [2].

*Sargassum filipendula* C. Agardh is a brown macroalgal species belonging to the widespread genus of brown seaweed, *Sargassum*. It is predominantly distributed throughout the tropical and subtropical basins of the Atlantic, Pacific, and Indian Oceans.

The massive proliferation of *Sargassum* spp. has culminated in the largest macroalgal bloom ever documented, commonly referred to as the Great Atlantic *Sargassum* Belt. In 2018, it reached a record-breaking peak, with an estimated 20 million metric tons of brown algae biomass spanning the Atlantic from the coast of West Africa through the Caribbean and the Gulf of Mexico to Brazil [5]. This phenomenon presents a multifaceted crisis, exerting profound ecological pressure on marine habitats while simultaneously posing severe ecological and socioeconomic threats to coastal economies that rely on tourism and artisanal fisheries. Valorizing this algal biomass may turn a significant environmental challenge into a ‘golden opportunity’ while simultaneously mitigating the impacts of coastal inundations. In recent years, several pathways for its valorization have been explored to take advantage of its rapid proliferation.

These include the production of bioenergy, such as liquid biofuels and biogas through anaerobic digestion [6,7], as well as applications in bioremediation for the removal of toxic compounds and elements, especially heavy metals from contaminated aqueous environments [8]. Furthermore, its high organic content makes it a viable candidate for soil amelioration, improving overall soil health and nutrient retention [9,10]. Most importantly, current research is shifting toward high-value applications, focusing on its development as a functional food and therapeutic agent. This interest is primarily driven by its rich profile of biologically active constituents, such as sulfated polysaccharides, flavonoids and phenolic acids, which exhibit significant antioxidant, immune modulation, and enzyme-inhibitory properties [11,12,13].Brown algae are known for the presence of sulfated polysaccharides, mainly fucoidans, which have numerous biological properties [14]. In addition to fucoidan, *S. filipendula* contains several classes of phytocompounds, such as carotenoids, phenols, glycolipids, and tannins (specifically phlorotannins) [15]. These compounds have been found to support the pharmacological properties of *S. filipendula*, highlighting its potential for development as a functional food or therapeutic agent [16].

Pharmacological studies have reported the broad-spectrum potential of *S. filipendula* against various infectious agents (bacteria, viruses, fungi, and parasites) and multiple non-infectious diseases and conditions, including anticancer, antioxidant, anti-aging, anti-osteoporosis, and immune-enhancing activities [15]. Significant pharmacological activities, such as anticancer activity, were found to be highest against cervical carcinoma cell lines among all eleven species compared in the study. Furthermore, the antioxidant activity of *S. filipendula*, specifically in terms of reducing power, was superior to the other eleven species tested and even exceeded that of ascorbic acid (used as a positive control) [17]. Similar to its antioxidant activity, the immunomodulatory activity of the *S. filipendula* constituents was effective, particularly in terms of NO production, which was comparable to the lipopolysaccharide (LPS) positive control used in the study [18]. *S. filipendula* extract was also found to modulate key targets, such as glycogen synthase kinase-3 beta (GSK-3β) and the mitochondrial-dependent apoptosis pathway. These interactions likely contribute to its anticancer efficacy against various cancer cells, including osteosarcoma, cervical carcinoma, prostate adenocarcinoma, and hepatocellular carcinoma [19]. The glycolipid-rich fraction also effectively reversed the drug resistance in Lucena-1 cells to vincristine.

Recent studies have highlighted the robust nutritional profile of *S. filipendula*, which is characterized by high contents of minerals, carbohydrates, proteins, essential amino acids, lipids, and dietary fiber [20]. The evaluated techno-functional properties, including water and oil absorption, emulsification, and foaming capacities, support the application of *S. filipendula* in various food products such as sausages, bakery items, soups, and sauces [20]. Recently, it was shown that the addition of *S. filipendula* flour to rice can enhance the functional potential and antioxidant capacity of rice flour in the study [21]. The robust nutritional profile and favorable techno-functional parameters, combined with multiple pharmacological activities, support the development of *S. filipendula* as a functional food.

Despite the presence of diverse bioactive phytochemical classes and significant pharmacological activities against both infectious and non-infectious diseases, as well as its ability to modulate critical biological targets and pathways, the development of *S. filipendula* remains in the preclinical stage, primarily limited to in vitro studies.

The absence of a comprehensive compilation of its pharmacological activities, phytochemicals, and molecular targets may hinder the systematic development of *S. filipendula* as a therapeutic agent. Therefore, the current review attempts to compile known pharmacological activities and phytochemical profiles along with existing research gaps. This review describes the isolation and identification of phytochemicals from *S. filipendula*, alongside their documented pharmacological activities. Furthermore, the review highlights the therapeutic potential of *S. filipendula* extracts by detailing their effectiveness, molecular targets, and pharmacological applications against both infectious diseases and critical non-infectious conditions, such as various types of cancer. Moreover, it suggests possible directions for transitioning *S. filipendula* components into a viable therapeutic or functional supplement against important diseases and conditions.

## 2. Literature Search Strategy

An electronic literature search was conducted across several databases, including PubMed, Web of Science, Scopus, and ResearchGate. The search utilized specific keywords and their combinations related to *S. filipendula* and its phytochemical and pharmacological properties, such as ‘*Sargassum filipendula*’, ‘*Sargassum filipendula* and phytocemical’, ‘*Sargassum filipendula* and antioxidant’, ‘*Sargassum filipendula* and anticancer’, ‘*Sargassum filipendula* and immunomodulation’, ‘*Sargassum filipendula* and antiosteoporosis’, ‘*Sargassum filipendula* and anti-aging’, ‘*Sargassum filipendula* and antibacterial’, ‘*Sargassum filipendula* and antiprotozoal’, and ‘*Sargassum filipendula* and antiviral’. Initial screening by title and abstract was performed for all retrieved articles. Finally, this narrative review includes English-language biomedical papers published through January 2026 that report phytochemical and pharmacological properties of *S. filipendula*.

## 3. Nutritional and Techno-Functional Profile of *S. filipendula*

A recent study on the nutritional characteristics of seaweed flour showed that *S. filipendula* has high protein, lipid, and carbohydrate contents, which were higher than those of commercially available *Sargassum* seaweeds (SC) [20].

The nutritional characteristics of *S. filipendula* flour prepared by two different methods, specifically freeze-drying or lyophilization (SFFD) and oven drying (SFOD), were evaluated. In both cases, high nutritional quality in terms of protein (16.81 ± 0.28 g/100 g), lipid (3.98 ± 0.27 g/100 g), and dietary fiber (78.42 ± 1.10 g/100 g) content was observed, which was higher in the case of SFFD. Generally, seaweeds are characterized by a high fiber content. For example, the dietary fiber contents of *Himanthalia elongata* and *Sargassum hemiphyllum* are up to 50.31% and 61.1%, respectively. However, *S. filipendula* exhibited a dietary fiber content of >78.42 g/100 g, which highlights its superior nutritional characteristics [20]. However, dietary fiber and other nutritional characteristics may vary with environmental conditions such as temperature, water salinity, nutrient availability, and sunlight exposure [22]. Furthermore, these values can be influenced by experimental procedures, particularly enzymatic–gravimetric methods [22,23]. The high protein content of *S. filipendula* flour contributes significantly to its nutritional value. However, comparable protein levels are found in other *Sargassum* species, such as *Sargassum horneri*, which is reported to contain up to 15%. Much like fiber content, protein levels vary depending on environmental conditions [24]. Studies have shown that the optimal protein content in seaweeds is typically achieved at the end of the winter season [25].

Similarly, high mineral content, including sodium (379.81 ± 7.28 mg/100 g), potassium (1557.77 ± 7.97 mg/100 g), calcium (2638.63 ± 9.75 mg/100 g), magnesium (919.53 ± 4.55 mg/100 g), iron (93.60 ± 1.16 mg/100 g), manganese (7.34 ± 0.01 mg/100 g), copper (0.30 ± 0.00 mg/100 g), and zinc (2.33 ± 0.01 mg/100 g), was observed [20]. These values were higher in SFFD for most minerals. The slightly lower levels of minerals observed in SFOD as compared to SFFD likely stem from the specific dehydration method. Because the seaweed was hung to dry and allowed to drip, soluble minerals such as iron were likely lost through leaching as water escaped from the tissue. Generally, seaweeds possess higher essential mineral contents compared to terrestrial vegetation [26]. Subject to further bioavailability studies, the high mineral content of *S. filipendula* may be utilized for populations with health conditions that restrict their intake of minerals from other nutrient-rich food sources. Further, amino acid analysis revealed a high total amino acid content (10.14 g/100 g) in SFFD. The aromatic amino acids (phenylalanine and tyrosine) were the most abundant, followed by leucine and lysine. Notably, it contained all essential amino acids in amounts close to or at par with the requirements suggested by the FAO for adults [20]. The high abundance of acidic amino acids, such as aspartic and glutamic acid, found in *S. filipendula* is consistent with reports on other seaweed species [27]. These amino acids may be responsible for the characteristic taste of algae and seafood products, contributing to the umami sensation [28]. The high concentration of these amino acids in *S. filipendula* makes it a promising candidate to replace monosodium glutamate (MSG) in food preparations, allowing for a label free of synthetic flavor additives. Similarly, *S. filipendula* flours (dried with different methods) after extraction with methanol showed high total phenolic content (TPC) through the Folin–Ciocalteu method, which was highest in SFFD (144.49 ± 0.87 mg of gallic acid equivalents per gram (mg GAE/g)), followed by SFOD (126.56 ± 1.44 mg GAE/g). These results suggest that *S. filipendula* flour prepared through the freeze-drying process has high potential for biological applications or as a functional food. A high TPC serves as a reliable indicator of the presence of biologically active phenolic compounds. In *S. filipendula*, this elevated TPC correlates with its biological efficacy, specifically its potent antioxidant activities [29].

Additionally, the physiochemical and techno-functional characteristics of the *S. filipendula* flours prepared through different drying methods and SC were analyzed to evaluate their properties and commercial prospects. The color of flours suggested that freeze-drying maintains the original color of the raw materials as compared with oven drying, resulting in the lighter and yellowish color. In freeze-drying, low temperature and pressure minimize the degradation of natural pigments, ensuring superior color retention. Consequently, chlorophylls and carotenoids are preserved in higher quantities and quality as compared to oven drying. Further, scanning electron microscopy results revealed that the SFFD exhibited superior preservation of the original cellular architecture, characterized by more intact cells and a porous, open structure. In contrast, SFOD displayed a significantly more collapsed and dense surface.

The techno-functional characteristics, such as the water solubility index, the water absorption index, oil holding capacity (OHC), emulsion capacity (EC), emulsion stability (ES), foam capacity, and stability (FC), were also evaluated and found to be supportive of its use in diverse food products such as sausages, bread, cakes, soups, and sauces. Notably, the water absorption index (7.52), EC (37.54%), ES (33.04%), and FC (14%) were found to be higher in SFFD [20]. Freeze-drying preserves a porous cellular structure and a larger surface area, providing more available sites for water absorption and enhancing emulsification capacity. Additionally, this process preserves the structural integrity required to retain gases released during foam formation, contributing to the superior gas retention observed in lyophilized samples.

The nutritional profile of *S. filipendula* supports its application in the development of fortified, nutrient-dense, and antioxidant-rich food products. Recently, partially substituting rice flour with 1.5% *S. filipendula* flour was shown to enhance the protein content, bioactive compounds, and antioxidant potential of the formulation, as verified through various antioxidant assays [30]. Further, incorporation of *S. filipendula* into the extrudate formulation significantly increased both hardness and moisture retention [30]. These structural changes are likely due to the high fiber and protein concentrations, which reinforce the molecular matrix and enhance the firmness of the extruded products [30]. A recent investigation into gluten-free cookie formulations demonstrated that a 1% inclusion of *S. filipendula* flour significantly enhanced the ash and moisture profiles [31]. Furthermore, this substitution yielded biscuits with a desirable soft texture and a darker aesthetic while simultaneously improving the protein digestibility and the total antioxidant capacity of the final product [31].

## 4. Phytochemicals of *S. filipendula*

The potential health benefits of *S. filipendula* serve as a primary driver for the phytochemical investigation of this species. While fucoidans (sulfated polysaccharides) are often credited with the majority of its biological properties, recent studies have highlighted a much broader spectrum of bioactive constituents, including phlorotannins (such as eckol, bifuhalol, and trifuhalol), carotenoids like fucoxanthin, and other diverse phytochemicals [15]. These compounds were identified through various studies utilizing various separation and analytical techniques [15].

The known pharmacological benefits of fucoxanthin, an allenic carotenoid found in edible brown algae, prompted the isolation of this compound from *S. filipendula*. A systematic extraction of various parts of *S. filipendula* was performed using dimethyl sulfoxide (DMSO), as DMSO is the powerful universal solvent used to penetrate plant cell membranes, ensuring the effective extraction of intracellular pigments [32]. This was followed by repeated partitioning with ethyl acetate to isolate the colored pigments, leveraging the intermediate polarity of ethyl acetate for effective separation. The residue was then re-extracted twice with acetone and further partitioned with hexane and distilled water to further refine the extract and remove the water-soluble impurities [33]. Further, fucoxanthin was isolated using column chromatography with a mobile phase consisting of n-hexane:ethyl acetate (6:4, *v*/*v*), which provides the specific polarity needed to move fucoxanthin specifically while leaving other pigments behind [34]. After isolation of pigment, the fucoxanthin was identified through high-performance liquid chromatography (HPLC), Fourier transform infra-red (FTIR) spectroscopy, and nuclear magnetic resonance (NMR) analysis. These methods confirmed the compound’s purity, functional groups, and molecular framework, respectively, providing definitive proof of its structure [34]. A high fucoxanthin content (~78%) was identified in the pigment extract of *S. filipendula* in this study. However, significant variations (ranging from 3.82 to 2927.98 μg/g dry weight) have been reported across different samples of *S. filipendula*, which can be further influenced by environmental conditions and extraction methodologies [35].

Later, antibacterial activity-guided fractionation was used to isolate and identify the specific phytochemicals responsible for the antimicrobial properties of *S. filipendula* [36]. Solvents of varying polarities were employed for the extraction to maximize antibacterial activity. This approach facilitates the recovery of a diverse range of phytochemicals, thereby optimizing the antimicrobial potential of the extracts. The nonpolar diethyl ether extract exhibited the highest antibacterial activity, suggesting that relatively nonpolar phytochemicals may be the primary drivers of the extract’s potency. Consequently, this fraction was selected for further phytochemical characterization using gas chromatography-mass spectroscopy (GC–MS), FTIR spectroscopy, and NMR analysis. These analytical methods identified five phytochemicals within the extract (Table 1), of which cis-7, cis-11-hexadecadien-1-yl acetate was the most abundant [36]. While identified phytochemicals comprised 31.6% of the diethyl ether extract, the remaining 68.4% remained uncharacterized due to the poor quality of these constituents in GC-MS analysis. Consequently, additional analysis is warranted to identify these minor constituents and evaluate their potential contributions to the extract’s biological activity [36].

In an earlier study, the chemical composition of the *S. filipendula* extract was characterized via GC-MS analysis. Dried algal biomass was extracted via a Soxhlet apparatus using a methanol/hexane (1:1) mixture until exhaustion [37]. The resulting extract was decolorized with activated charcoal, filtered, and concentrated under reduced pressure at 30–45 °C (using a rotatory evaporator). The dried residue was reconstituted in 3 mL of DMSO. The helium served as the carrier gas at a constant flow rate of 1 mL/min in the GC-MS analysis. Analysis revealed the presence of 23 compounds belonging to various chemical classes, including fatty acids, hydrocarbons, aldehydes, ketones, terpenes, alcohols, esters, and phenols (Table 1).

Primarily, phlorotannin compounds were identified from the phlorotannin extract of *S. filipendula* [16]. The washed, dried, and ground raw material underwent successive extractions for 12 h in acetone with magnetic stirring at room temperature to obtain the phlorotannins. The HPLC-electrospray ionization (ESI)-MS analysis used in the identification of three possible compounds from the phlorotannins fraction of *S. filipendula* extract (PESF). The compounds were identified as Eckol, Bifuhalol, and Trifuhalol. The phlorotannin extracts were further analyzed for different pharmacological activities, including antioxidant and antiaging [16].

Recently, given that seaweeds are rich in glycolipids and considering the importance of these compounds in reversing cancer drug resistance, the glycolipids in *S. filipendula* were identified. Powdered *S. filipendula* was extracted using a chloroform-methanol solution, then further fractionated via silica gel column chromatography and eluted with solvents of increasing polarity. Finally, eight fractions identified as glycolipid-positive were subjected to UV-Visible, FTIR, NMR, and LC-MS analyses to characterize their components [38]. A total of ten sulfoquinovosylglycerol-type compounds were identified, six (1-mono-2-O-hexadecanoyl-3-O-(6-sulfoquinovopyra-nosyl)-glycerol, 1-mono-2-O-(9,12,15-octadeca-tri-enoyl)-3-O-(6-sulfoquinovopyranosyl)-glycerol, 1-O-(9-heptadecenoyl),2-O-hexadecanoyl-3-O-(6-sulfoquinovopyra-nosyl)-glycerol, 1-O-octadecanoyl,2-O-hexadecanoyl-3-O-(6- sul-foquinovopyra-nosyl)-glycerol, 1-O-docosanoyl,2-O-(9-hexadecenoyl)-3-O-(6- sulfoquinovopyranosyl)-glycerol, 1-O-docosanoyl,2-O-hexadecanoyl-3-O-(6- sulfoquinovopyranosyl)-glycerol) of which are reported for the first time and four of which are reported to be present in the species *S. vulgare* (Table 1).

Recently, the residual biomass of *S. filipendula* remaining after the extraction of alginates and other polysaccharides was characterized to identify its chemical composition [15]. The analysis revealed that the residual biomass contains, on average: proteins (7.36% ± 0.0031), lipids (1.11% ± 0.0011), ash (20.51% ± 0.0049), and moisture (13.59% ± 0.0081). Furthermore, it was found to be rich in insoluble fiber (27.21% ± 0.0184) and total soluble fiber (23.04% ± 0.0191), with a residual alginate content of 5.89% ± 0.0035. Deep eutectic solvent systems (DESS) were utilized to extract phenolic compounds across 24 different systems. Among these, the combinations of N,N-(dimethylamino)-ethanol with benzyl alcohol at a 1.30 to 1 ratio (DMAE-BzOH) and N,N-(dimethylamino)-ethanol with 1,3-propanediol (DMAE-1,3-PDO) at a 1.83 to 1 ratio were identified as the most efficient solvents for the extraction of bioactive compounds. These results showed 8.79 mg gallic acid equivalent (GAE)/g of TPC and 9.24 mg phloroglucinol equivalent (PGE)/g of total tannin compounds (TTC). In comparison, DMAE-1,3-PDO extraction yielded 6.20 mg GAE/g of TPC and 11.34 mg PGE/g of TTC. The content of TPC was found to be increased in the DESS with the addition of water up to 30%; after that it drops, which suggests the controlled hydration modulates the viscosity of DESS by weakening the intermolecular interactions between the hydrogen bond donor and hydrogen bond acceptor. This reduction in viscosity enhances mass transfer and optimizes extraction yields. Conversely, excessive water content can destabilize the DESS, leading to a total loss of the characteristic intermolecular interactions [15].

**Table 1:** Phytochemicals reported in *S. filipendula*.

Sr. No.	Compound Name	Amount	Type of Extract/Fraction	Type of Compound	References
1	13-Octadecenoic acid, methyl ester	2.09%	Diethyl ether extract	Ester	[36]
2	E-11-Hexadecenal	4.55%	Diethyl ether extract	Aldehyde	[36]
3	cis-7, cis-11-Hexadecadien-1-yl acetate	21.27%	Diethyl ether extract	Ester	[36]
4	Z,E-7,11-Hexadecadien-1-yl acetate	1.23%	Diethyl ether extract	Ester	[36]
5	9,12-Octadecadienoyl chloride, (Z, Z)-	2.16%	Diethyl ether extract	Acyl chloride	[36]
6	11-octadecenoic acid methyl ester	2.09%	Diethyl ether extract	Ester	[36]
7	1-mono-2-O-hexadecanoyl-3-O-(6-sulfoquinovopyranosyl)-glycerol	NP	fractions enriched in glycolipids	Glycolipids	[38]
8	1-mono-2-O-(9,12,15-octadecatrienoyl)-3-O-(6-sulfoquinovopyranosyl)-glycerol	NP	fractions enriched in glycolipids	Glycolipids	[38]
9	1-O-tetradecanoyl,2-O-hexadecanoyl-3-O-(6-sulfoquinovopyranosyl)-glycerol	NP	fractions enriched in glycolipids	Glycolipids	[38]
10	1,2-di-O-hexadecanoyl-3-O-(6-sulfoquinovopyranosyl)-glycerol	NP	fractions enriched in glycolipids	Glycolipids	[38]
11	1-O-(9-heptadecenoyl),2-O-hexadecanoyl-3-O-(6-sulfoquinovopyranosyl)-glycerol	NP	fractions enriched in glycolipids	Glycolipids	[38]
12	1-O-(9-octadecenoyl),2-O-hexadecanoyl-3-O-(6-sulfoquinovopyranosyl)-glycerol	NP	fractions enriched in glycolipids	Glycolipids	[38]
13	1-O-octadecanoyl,2-O-hexadecanoyl-3-O-(6-sulfoquinovopyranosyl)-glycerol	NP	fractions enriched in glycolipids	Glycolipids	[38]
14	1-O-nonadecanoyl,2-O-hexadecanoyl-3-O-(6-sulfoquinovopyranosyl)-glycerol	NP	fractions enriched in glycolipids	Glycolipids	[38]
15	1-O-docosanoyl,2-O-(9-hexadecenoyl)-3-O-(6-sulfoquinovopyranosyl)-glycerol	NP	fractions enriched in glycolipids	Glycolipids	[38]
16	1-O-docosanoyl,2-O-hexadecanoyl-3-O-(6-sulfoquinovopyranosyl)-glyceroly	NP	fractions enriched in glycolipids	Glycolipids	[38]
17	Eckol	NP	polyphenols crude extract	Phlorotannins	[16]
18	Bifuhalol	NP	polyphenols crude extract	Phlorotannins	[16]
19	Trifuhalol	NP	polyphenols crude extract	Phlorotannins	[16]
20	Fucoxanthin	78.9% ^#^	Pigment extract	Carotene	[34]
21	β-carotene	NP	Pigment extract	Carotene	[33]
22	Oleic Acid	1.07% *	Ethyl acetate fraction of methanol extract	Fatty acid	[37]
23	Propiolic acid, 3-(1-hydroxy-2-isopropyl-5-methylcyclohexyl)-	0.46% *	Ethyl acetate fraction of methanol extract	Fatty acid	[37]
24	9-Hexadecenoic acid	0.92% *	Ethyl acetate fraction of methanol extract	Fatty acid	[37]
25	n-Hexadecanoic acid	0.76% *	Ethyl acetate fraction of methanol extract	Fatty acid	[37]
26	Cholestan-3-ol, 2-methylene-, (3β,5α)-	4% *	Ethyl acetate fraction of methanol extract	Terpene	[37]
27	Dihydroxanthin	0.3% *	Ethyl acetate fraction of methanol extract	Ketone	[37]
28	5H-Cyclopropa[3,4]benz[1,2-e]azulen-5-one, 9-(acetyloxy)-3- [(acetyloxy)methyl]-1,1a,1b,4,4a,7a,7b,8,9,9a-deca	2.3% *	Ethyl acetate fraction of methanol extract	Ketone	[37]
29	5H-Cyclopropa[3,4]benz[1,2-e]azulen-5-one, 4,9,9a-tris (acetyloxy)-3-[(acetyloxy)methyl]-1,1a,1b,4,4a,7a,7b,8,9,	1.4% *	Ethyl acetate fraction of methanol extract	Ketone	[37]
30	5H-Cyclopropa[3,4]benz[1,2-e]azulen-5-one, 9a-(acetyloxy)- 1,1a,1b,4,4a,7a,7b,8,9,9a-decahydro-4a,7b,9-trihyd	NP	Ethyl acetate fraction of methanol extract	Ketone	[37]
31	Ethanol, 2-(9-octadecenyloxy)-, (Z)-	3.07% *	Ethyl acetate fraction of methanol extract	Alcohol	[37]
32	13-Heptadecyn-1-ol	5.5% *	Ethyl acetate fraction of methanol extract	Alcohol	[37]
33	1-Heptatriacotanol	1.38% *	Ethyl acetate fraction of methanol extract	Alcohol	[37]
34	Acetic acid, 2-(2-acetoxy-2,5,5,8a-tetramethyldecalin-1-yl)-	3.8% *	Ethyl acetate fraction of methanol extract	Aldehyde	[37]
35	2-[4-methyl-6-(2,6,6-trimethylcyclohex-1-enyl)hexa-1,3,5- trienyl]cyclohex-1-en-1-carboxaldehyde	8% *	Ethyl acetate fraction of methanol extract	Aldehyde	[37]
36	Phen-1,4-diol, 2,3-dimethyl-5-trifluoromethyl-	NP	Ethyl acetate fraction of methanol extract	Phenol	[37]
37	Cyclopropanebutanoic acid, 2-[[2-[[2-[(2-pentylcyclopropyl) methyl]cyclopropyl]methyl]cyclopropyl]methyl]-, methyl ester	3.8% *	Ethyl acetate fraction of methanol extract	Ester	[37]
38	9,12,15-Octadecatrienoic acid, 2,3-dihydroxypropyl ester, (Z,Z,Z)-	0.46% *	Ethyl acetate fraction of methanol extract	Ester	[37]
39	Docosahexaenoic acid, 1,2,3-propanetriyl ester	0.61% *	Ethyl acetate fraction of methanol extract	Ester	[37]
40	Dasycarpidan-1-methanol, acetate (ester)	12.2% *	Ethyl acetate fraction of methanol extract	Ester	[37]
41	Retinoic acid, methyl ester	3.3% *	Ethyl acetate fraction of methanol extract	Ester	[37]
42	Dasycarpidan-1-methanol, acetate (ester)	2% *	Ethyl acetate fraction of methanol extract	Ester	[37]
43	Acetic acid, 3-hydroxy-4,4,10,13-tetramethyl-7-oxo- 2,3,4,7,8,9,10,11,12,13,14,15,16,17-tetradecahydro-1H-cyclo	37.2% *	Ethyl acetate fraction of methanol extract	Hydrocarbon	[37]
44	Octadecanal, 2-bromo-	32.5% *	Ethyl acetate fraction of methanol extract	Hydrocarbon	[37]

NP: not provided; ^#^: among extracted pigments; *: percent peak area.

**Figure 1:**
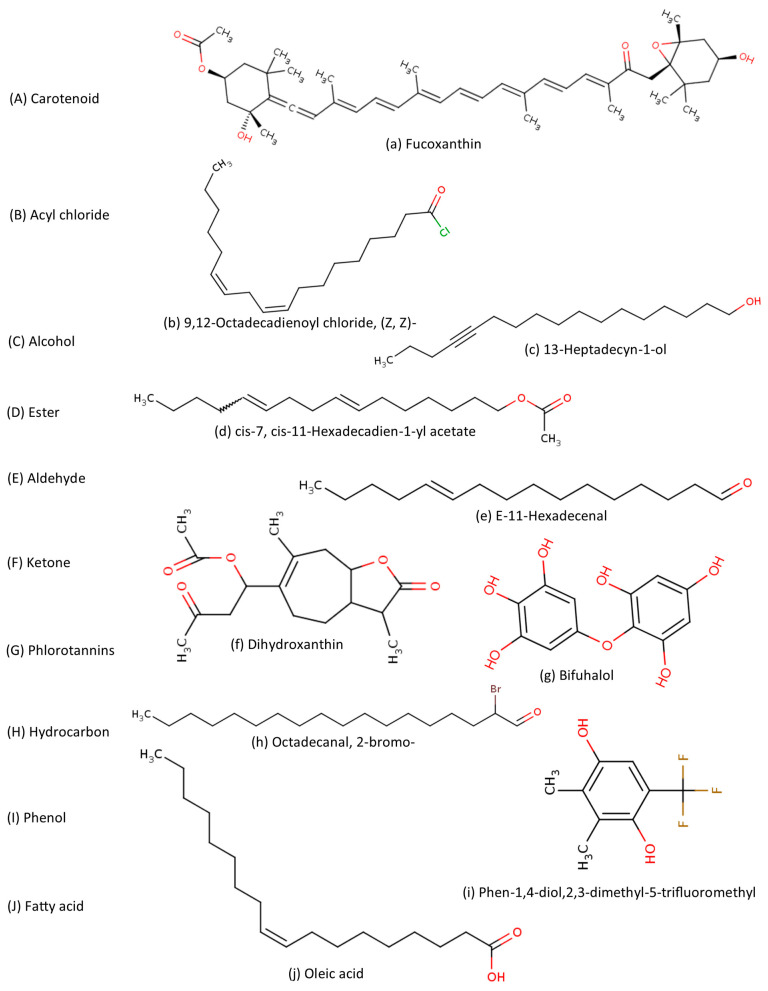
Representative chemical structures of major phytochemical classes identified in *Sargassum filipendula*. A single characteristic structure is shown for each category.

Researchers have revealed the diverse phytochemical profile of *S. filipendula* by utilizing various solvents and methodologies tailored to target phytochemicals or specific biological properties of interest (Figure 1). Antibacterial-guided selection among methanol, ethanol, and diethyl ether revealed that the relatively nonpolar diethyl ether facilitated the separation and identification of primarily ester and aldehyde classes [36]. In contrast, a biphasic combination of polar and nonpolar solvents (methanol/hexane) enabled the identification of a broader range of phytochemicals, including alcohols, aldehydes, phenols, esters, hydrocarbons, fatty acids, and terpenes, within the extracts [37]. Specific classes of compounds, such as glycolipids, were isolated using a chloroform-methanol solvent system [38]. Furthermore, the carotenoid fucoxanthin was successfully separated and identified through a systematic, sequential extraction process utilizing an array of solvents of varying polarities. Overall, these studies have established a unique phytochemical profile for *S. filipendula*, which supports its diverse pharmacological activities (Table 1). However, several phytochemicals in these studies remained uncharacterized due to the low concentration and poor resolution of these constituents in GC-MS analysis [36]. Further analytical investigation is therefore necessary to characterize these minor constituents and determine their synergistic or individual contributions to the extract’s pharmacological effects.

## 5. Pharmacological Activities of *S. filipendula*

*S. filipendula* possesses a robust nutritional profile characterized by high concentrations of minerals, proteins, lipids, dietary fibers, and sulfated polysaccharides such as fucoidans, as well as a diverse array of phytochemicals like polyphenols. These constituents, especially the phytochemicals, provide the biochemical foundation for its significant biological potential. Numerous studies utilizing experimental models have demonstrated that *S. filipendula* exhibits diverse pharmacological properties, including antioxidant, anticancer, anti-aging, antimicrobial, and immunomodulatory activities.

### 5.1. Antioxidant Activity

Antioxidant activity is important due to the high demand for it in both the food and pharmaceutical industries. Natural antioxidants are preferred in comparison with synthetic ones in both the food and pharmaceutical industries [39,40]. Fucoidan present in the *S. filipendula* is the important natural antioxidant that is the main component considered responsible for different health-promoting activities of *S. filipendula* [41]. However, phytocompounds such as fucoxanthin and phlorotannins present in *S. filipendula* also showed strong antioxidant activities [33].

#### In Vitro Antioxidant Activity of *S. filipendula* Extract

In a screening study, sulfated polysaccharides from 11 species of tropical marine algae, including *S. filipendula*, were evaluated for their antioxidant and anticancer activities. The significant activity was not observed in radical scavenging assays. However, in the reducing power assay, sulfated polysaccharides from *S. filipendula* exhibited the highest antioxidant activity among the 11 species studied, even surpassing the performance of the positive control, ascorbic acid [17]. According to the literature, heterofucans isolated from other brown seaweeds, such as *Canistrocarpus cervicornis*, have also demonstrated significant antioxidant activities across various experimental methods [42]. The potent antioxidant activity observed in the initial screening prompted the further isolation of heterofucan fractions from *S. filipendula* and the evaluation of their additional biological properties.

In an initial study, the proteolytic digestion of *S. filipendula*, followed by sequential acetone precipitation, yielded five distinct heterofucans [19]. These five heterofucans (SF0.5, SF0.7, SF1, SF1.5, and SF2) showed increasing concentrations of total sugars and sulfate. The antioxidant properties of all five heterofucans were evaluated using various assays, including total antioxidant capacity (TAC), reducing power, ferrous ion chelating activity, and hydroxyl and superoxide radical scavenging activities. In the TAC assay, SF1 exhibited the highest activity at 90.7 ascorbic acid equivalents (AAE), whereas SF2 showed the minimum activity at 9.6 AAE (Table 2). Regarding radical scavenging activities, SF0.7 exhibited the highest hydroxyl and superoxide radical scavenging percentages at a concentration of 0.5 mg/mL. All five heterofucans exhibited significant dose-dependent ferrous ion chelating activity, with SF2 displaying the highest potency. Results indicated that the sulfate concentration, which was highest in SF2, may be critical for ferrous ion chelation; this observation is well-supported by the existing literature [41]. Similarly, SF1 showed the highest reducing power, which was comparable to that of ascorbic acid, the reference compound used in this experiment [19].

Fucoxanthin is considered a pharmacologically active compound responsible for various biological properties of brown algae, including antioxidant and anti-inflammatory activities [43]. Consequently, the antioxidant activity of fucoxanthin isolated from *S. filipendula* was evaluated using the DPPH radical scavenging assay [33]. In this experiment, the antioxidant activity of fucoxanthin was found to be comparable to β-carotene, one of the most common and well-known carotenoids. Notably, the antioxidant activity of fucoxanthin isolated from *S. filipendula* in the current study was significantly higher than that reported for fucoxanthin isolated from *Sargassum oligocystum* [35]. The potent antioxidant activity of fucoxanthin prompted an investigation into its anticancer properties, which were evaluated in subsequent experiments [33].

Initially, the antioxidant activity of sulfated carbohydrate (fucoidan) from the *S. filipendula* was studied using the DPPH assay. The fucoidan extract was prepared from the dry powder of *S. filipendula*. The 0.1 M HCl was used to hydrolyze the cell wall, and different solvents were used to prepare the fucoidan extract. The yield of fucoidan was 4.67%, and the information on fucoidan functional groups was obtained using FTIR, which showed that the fucoidan samples obtained from the extraction process have sulfate ester O=S=O (sym) and O=S-O (asym) in the absorption area of 1260.39 cm^−1^ and 1027.83 cm^−1^, respectively. Meanwhile, the absorption area of 822.79 cm^−1^ indicates the position of sulfate, which binds to the fucoidan chain in an equatorial position. The antioxidant activity of crude fucoidan extract, sulfonated fucoidan (using sulfur trioxide-pyridine complex reagent), and standard fucoidan were studied. The sulfur content may increase the antioxidant activity of fucoidan, as the presence of sulfate groups in polysaccharide molecules is able to activate hydrogen atoms in anomeric carbon. The antioxidant activity of crude fucoidan extract from *S. filipendula* was more in the case of distilled water solvent compared to DMSO in the study [41]. The antioxidant activity of fucoidan was increased after sulfonation, and the highest antioxidant activity was observed in the case of standard fucoidan, which suggests that the presence of impurity in the extracted fucoidan may limit antioxidant activity.

Later, considering the fact that the contents of active compounds and antioxidant activity may be influenced by the extraction conditions, such as the extraction method, temperature, time, and solvent concentration, the researchers measured the fucoidan content and antioxidant activity by systematically varying the HCl solvent concentration (0.01 M, 0.03 M, and 0.05 M) and extraction time (10, 15, and 20 min) within an ultrasonic-assisted extraction system [44]. Under the tested conditions, the highest recorded fucoidan content was 6.07%, which resulted specifically from the 0.03 M HCl concentration and 15 min extraction time during Ultrasound-Assisted Extraction. The same condition also exhibited the highest antioxidant activity in the study (Table 2). The fucoidan’s antioxidant activity observed in this study was higher than that of fucoidan extracted from *S. polycystum* in an earlier study [44]. This difference indicates that the species type significantly influences not only the fucoidan yield but also its antioxidant potential [44].

Abiotic factors, such as ultraviolet (UV) radiation, can significantly impact the biology and metabolic activities of seaweeds. Consequently, these environmental stressors alter the seaweed’s phytochemical profile and subsequent biological activities. The impact of UV radiation as a common environmental effector was evaluated regarding the biological activity of *S. filipendula* extracts [29]. Specifically, the influence of UVA and UVB exposure on antioxidant activities was investigated. The ten-day exposer to UVA, UVB along with photosynthetically active radiation (PAR) was analyzed along with the control group. Oxidative DNA damage in *S. filipendula* was evaluated by quantifying cyclobutane pyrimidine dimers (CPDs). The results revealed a significant increase in CPDs only in the UVB-exposed group, suggesting that high-energy UVB radiation exerts a potent DNA-damaging effect. The antioxidant activities evaluated through DPPH, ABTS, FRAP, and ferrous ion chelating ability assays revealed effective dose dependent antioxidant properties across all groups. However, the highest activity was observed in the control group, which was not subjected to UV exposure followed by UVA and UVB groups. The TPC followed the same pattern as the antioxidant activities in *S. filipendula*, with values ranked as: Control > UVA > UVB. Overall, it can be concluded that UV exposure caused an adverse impact on *S. filipendula*. This effect was more severe in the UVB groups, which exhibited significant damage that resulted in lower phenolic content and, subsequently, reduced antioxidant activities.

Recently, the researcher extracted fucoidans and other bioactive compounds such as polyphenols (phlorotannins) from the *S. filipendula*. The washed, dried, and ground raw material underwent successive extractions for 12 h in acetone with magnetic stirring at room temperature to obtain the phlorotannins. Fucoidan was subsequently extracted following the previously described method [16]. Purified fucoidan, crude fucoidan, and crude phlorotannin extracts were evaluated with the commercial fucoidan for antioxidant activities. Different biological properties, including antioxidant activities of extracts and corresponding compounds, were analyzed. The antioxidant activity of extracts was studied with different methods, including lipid peroxidation of methyl linoleate, superoxide radical, ABTS radical, hydroxyl radical, and DDPH scavenging activities [16]. The antioxidant efficacy of the crude extracts was demonstrated by their capacity to scavenge radicals and inhibit lipid peroxidation, as quantified through thiobarbituric acid reactive substances (TBARS) and conjugated diene hydroperoxide (CDH) assays. Crude extracts had higher antioxidant activities compared with purified extracts in all methods used in the study (Table 2). Notably, the antioxidant activity of PESF in DPPH radical scavenging was superior to that previously reported for the polyphenol extract of *S. binderi* and the phlorotannin extract of *Fucus vesiculosus* across various solvents, including water, methanol, ethanol, ethyl acetate, and acetone [16,45].

In a recent nutritional study of *S. filipendula*, the antioxidant activity of freeze-dried (SF-FD) and oven-dried *S. filipendula* (SF-OD) was evaluated along with commercially available *S.* seaweed (SC) [20]. *S. filipendula* flours produced through both drying methods were analyzed for total phenolic compound content before conducting antioxidant activity testing. Extraction was performed using methanol in an ultrasonic bath (40 min, 30 °C), and total phenolic content was determined through the Folin–Ciocalteu method. Antioxidant assays using DPPH, ABTS, and FRAP methods revealed significant activities, which were highest in SF-FD, followed by SF-OD, while the minimum activity was observed in SC. The phenolic content was also found to follow the same ranking order as the antioxidant activities. The existing literature corroborates the findings; consistent with the observations in *S. filipendula*, the antioxidant capacity of *Fucus vesiculosus* has been shown to correlate with the concentration of bioactive phenolic compounds, present in the samples [46]. It can be concluded that the potent antioxidant activity observed in *S. filipendula* is a multifaceted result of the synergistic interaction between its primary bioactive constituents, which specifically include sulfated polysaccharides, carotenoids such as fucoxanthin, and various polyphenolic compounds like phlorotannins. Through the optimization of these specific contents, a measurable increase in the overall antioxidant capacity of the *S. filipendula* extract was achieved, thereby highlighting the importance of standardized extraction protocols. While the efficacy of the extract has been rigorously validated through established in vitro chemical models, including DPPH or ABTS free radical scavenging, ferric reducing antioxidant power, metal chelation, and lipid peroxidation inhibition, these assays do not fully account for complex biological environments. Currently, the biologically relevant effects of *S. filipendula* on enzymatic antioxidants such as superoxide dismutase (SOD), catalase (CAT), ascorbate peroxidase (APX), monodehydroascorbate reductase (MDHAR), dehydroascorbate reductase (DHAR), glutathione reductase (GR), and glutathione peroxidase (GPx) remain largely unexplored. Elucidating these intracellular enzymatic pathways is essential for characterizing the true therapeutic and pharmacological potential of *S. filipendula*, and consequently, these investigations are strongly recommended as a priority for future research.

### 5.2. Antiaging Activity of S. filipendula Extract

Antiaging research has gained importance as a scientific field due to the increasing prominence of population aging. Natural products have shown their anti-aging potential in various studies and are preferred because long-term medication is often required [47,48]. Furthermore, these natural compounds are generally considered safer than their synthetic counterparts.

**Figure 2:**
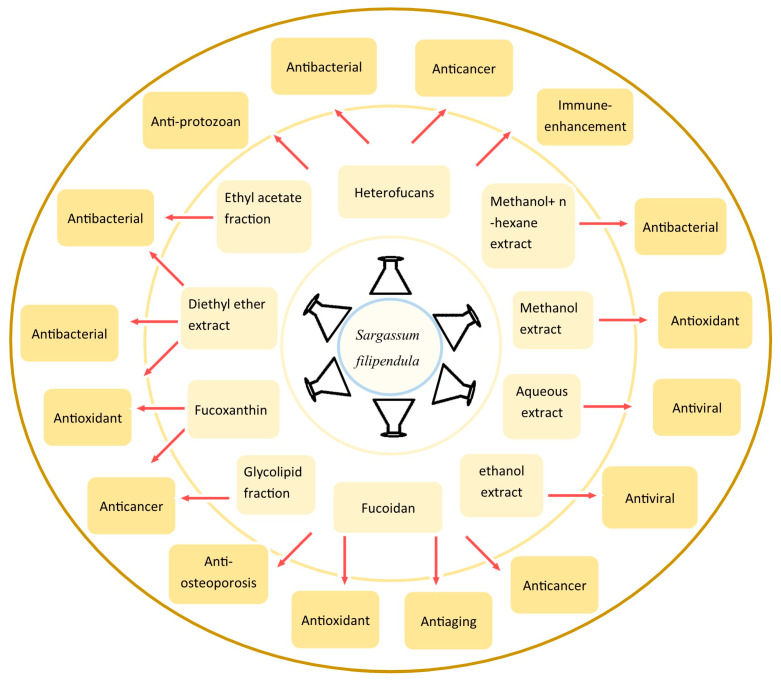
Different pharmacological activities of *S. filipendula* through different extract and forms reported in the literature.

After evaluating the antioxidant potential of PESF (a polyphenol-rich fraction) and FESF (the fucoidan extract of *S. filipendula*), their anti-aging activities were assessed via the inhibition of matrix metalloproteinases (MMPs) [16]. These enzymes, specifically collagenase and elastase, are primary drivers of skin aging, as they catalyze the degradation of type I collagen and elastic fibers, leading to a loss of structural elasticity and the formation of wrinkles [49]. To evaluate the anti-aging potential of the selected fractions, their inhibitory effects on collagenase and elastase were investigated using standardized enzymatic inhibition assays [49]. The results indicated that both extracts exhibited potent inhibitory effects on these proteolytic enzymes (Figure 2 and Table 2). Notably, the anti-elastase activity of both extracts was comparable to that of the positive control, suggesting a high degree of efficacy in preserving elastin fibers. In contrast, the anti-collagenase assay revealed that PESF possessed superior inhibitory strength compared to FESF (Table 2). This heightened efficacy in PESF may be attributed to the synergistic effects of the phenolic compounds present within the fraction, particularly phlorotannins. These results align with a recent study demonstrating that brown algal phlorotannins mitigate skin aging through the targeted inhibition of MMPs [50].

### 5.3. Anticancer Activity of S. filipendula

The global incidence of cancer continues to rise, and current demographic projections indicate that the number of new cases is expected to reach 35 million by the year 2050 [51]. This alarming trend underscores the urgent need for effective preventive strategies and novel therapeutic interventions that can mitigate the long-term impact of this disease. Natural products are considered an effective reservoir of bioactive compounds that can be developed into anticancer agents, which have already demonstrated potent anticancer activities [52,53].

Following the demonstration of the superior reducing power of *S. filipendula* sulfated polysaccharides among 11 tropical seaweed species, their anticancer activity was evaluated against the HeLa cervical carcinoma cell line [17]. The dose-dependent inhibition of HeLa cells was observed through the 3-(4,5-Dimethylthiazol-2-yl)-2,5-diphenyltetrazolium bromide (MTT) assay, which was highest for *S. filipendula* among all tested seaweed species. These encouraging anticancer results led to subsequent mechanistic analyses of the *S. filipendula* heterofucans [54].

Later, the heterofucan termed SF1.5, isolated from *S. filipendula*, as previously described, was evaluated for its anticancer activity against the HeLa cervical carcinoma cell line [54]. Dose-dependent inhibition of HeLa cells was observed through MTT assay. The highest inhibition observed in this study was 72.5% (at 2.0 mg/mL), which was superior to the activities reported for fucans isolated from other species, such as *Sargassum kjellmanianum* and *Sargassum stenophyllum* [54]. Inducing apoptosis is a common pathway utilized by therapeutics to manifest their anticancer potential, leading to the targeted elimination of malignant cells [55,56]. A significant increase in apoptotic cells was observed following treatment with SF1.5, suggesting that it induces apoptosis. However, Western blot (WB) analysis revealed that aspartate-specific cysteinyl proteases (caspases) 3 and 9 are not involved in SF1.5-mediated activity, as their expression levels remained unaltered after treatment. Similarly, the SF1.5 did not alter the phosphorylation of NF-κB, ERK, and p38, which rules out the role of the NF-κB and MAPK pathways in the activity. These results suggest that SF1.5-induced apoptosis occurs independently of the caspase, NF-κB, and MAPK pathways. However, SF1.5 induces the dephosphorylation (activation) of glycogen synthase kinase-3 beta (GSK-3β). Since GSK-3β dephosphorylation is associated with apoptosis, this suggests that SF1.5 likely triggers apoptosis primarily through GSK-3β activation. However, treatment with lithium chloride, a GSK-3-specific inhibitor, failed to attenuate SF1.5-induced apoptosis. This indicates that GSK-3 activation is not essential for the apoptotic effect of SF-1.5v in HeLa cells. However, bifunctional role of GSK-3β in cell death pathways is reported. These findings are consistent with results reported for fucan from *Fucus vesiculosus*, which promotes GSK-3β dephosphorylation; however, this effect is not involved in fucan-induced cell death in human HS-Sultan cells. The mitochondrial pathway can activate a caspase-independent form of programmed cell death. This process involves mitochondrial outer membrane permeabilization, which releases intermembrane space proteins into the cytosol. Among these is the apoptosis-inducing factor (AIF), which translocates to the nucleus to trigger apoptotic morphology independently of caspase activity. Cytosolic levels of AIF increased upon exposure to SF1.5, suggesting that the primary mechanism of SF1.5-induced cell death involves the mitochondrial release of AIF into the cytoplasm. Several phytochemicals, including cerulenin, berberine, and resveratrol, have been reported to induce the release of AIF from the mitochondria to the cytosol, thereby exerting potent anticancer effects across various malignancies [57,58].

Later, after demonstrating the antioxidant activity of five distinct heterofucans isolated from *S. filipendula*, the anticancer potential of all five heterofucans was studied on HeLa (cervical carcinoma), PC3 (prostate adenocarcinoma), and HepG2 (hepatocellular carcinoma) cell lines [19]. Antioxidant activity can support the anticancer activity by neutralizing reactive oxygen species (ROS) and maintaining cellular redox homeostasis. All heterofucans exhibited dose-dependent growth inhibition across all tested cell lines, except for SF0.7, which showed no inhibitory effect on HeLa cells. While SF1 and SF1.5 were classified as high-activity heterofucans with IC_50_ values of 15.69 μM and 13.83 μM, respectively. Based on IC_50_ values, the heterofucans in this study exhibited higher anticancer activity against HepG2 and HeLa cells than those previously reported for *Cladosiphon okamuranus* and *Ascophyllum nodosum*, respectively [19]. The variation in anticancer efficacy suggests that activity depends on the specific cell line tested. Such divergence implies a mechanism of action characterized by functional selectivity, likely influenced by the distinct genetic and proteomic landscapes of the tested malignancies. Consequently, further study into the underlying mechanisms is required to optimize the effect of these heterofucans.

Later, following the observation of potent antioxidant activity, the anticancer potential of fucoxanthin isolated from *S. filipendula* was subsequently evaluated against HeLa cells. Apoptosis induction in HeLa cells was investigated using the terminal deoxynucleotidyl transferase dUTP Nick-End Labeling (TUNEL) assay following treatment with varying doses of fucoxanthin isolated from *S. filipendula* [33]. The potent anticancer activity of fucoxanthin was apparent through the induction of apoptosis in nearly 100% of cells at dosages of 50 ppm and above (up to 100 ppm). The apoptotic induction observed following fucoxanthin treatment was not only consistent with, but significantly superior to, the effects previously reported for the *S. filipendula* heterofucan (SF1.5) [54].

The literature indicates that the molecular weight fractions of fucoidans significantly affect their overall biological activity, including anticancer activity [59]. To investigate the influence of the molecular weight of fucoidans on biological activity, crude extract (CFSF) and low (LFSF), medium (MFSF), and high molecular weight fractions (HFSF) of fucoidan isolated from *S. filipendula* were evaluated for their anticancer activity against osteosarcoma, alongside fucoidan from *Fucus vesiculosus* (CFFV) [60].

The neutral sugar (fucose and galactose), uronic acid, and sulfate contents of the fucoidans were quantified prior to evaluating their anticancer activities. The sulfate content was highest in the crude fucoidan isolated from *Fucus vesiculosus*; however, all other parameters were highest in the medium molecular weight fractions of fucoidan from *S. filipendula*.

MG63 human osteosarcoma cells were utilized to evaluate the efficacy of selected fucoidan fractions against osteosarcoma, one of the most frequently occurring primary bone cancers [60]. MG-63 cells are among the most widely utilized cell lines for evaluating anticancer activity against osteosarcoma. These cells maintain a stable phenotype over multiple passages, making them a reliable in vitro model for assessing the biocompatibility and biofunctionality of both implant materials and novel therapeutics [61]. Dose-dependent reductions in mean cell metabolic activity, mean cell DNA content, focal adhesion formation, and cell proliferation were observed across all fucoidan types; however, CFFV and the HMW fraction exhibited the most potent inhibitory effects [60]. Cell attachment measured by metabolic activity and DNA content showed the lower cell attachment by HFSF compared with MFSF and LFSF. In Giemsa staining, dose-dependent alterations in the morphology and death of cells were observed with treatment of fucoidan fractions from *S. filipendula*. Further, ultrastructure examination of MG63 cells showed that the fucoidan-treated cells exhibited actin-like filament condensation within the cytosol. The mitochondria appeared dense with swollen cristae, which progressed to disintegration in more severely damaged cells. Features of autophagy (autophagocytosis) were observed in select treated cells, characterized by dense granulated structures or vesicles sequestering cellular material. Furthermore, cell swelling, ruptured membranes, and cytoplasmic leakage were common features across all fucoidan treatments. Morphological observations revealed the presence of membrane blebbing in a subset of cells treated with MFSF and HFSF. Overall, the toxic effects were more in the CFSF, HFSF, and MFSF than in the LFSF [60].

Annexin V/PI, TUNEL, and cytochrome c staining confirmed that *F. vesiculosus* fucoidan induces apoptosis-like cell death, whereas *S. filipendula* fucoidans exhibit features of necrosis-like cell death. These distinct results demonstrate a clear difference in the mechanism of action between the fucoidans of these two species. Notably, the induction of regulated necrotic cell death is increasingly recognized as a strategy in oncology, particularly for its potential to bypass the mechanisms of drug-resistant cancer cells [62].

Mitochondrial integrity in MG63 cells was assessed via JC-1 staining following exposure to fucoidan. All fucoidan variants from *S. filipendula* induced mitochondrial membrane depolarization, evidenced by a shift toward a higher green-to-red fluorescence ratio, with the HFSF fraction exhibiting the greatest potency. These results are consistent with previous studies, suggesting that the mitochondrial plays an important role in the anticancer activity of heterofucans derived from *S. filipendula* [54].

Immunostaining was performed to evaluate the cellular localization of various fucoidans in MG63 cells. Crude fucoidan from *S. filipendula* demonstrated extensive cytosolic penetration, whereas *F. vesiculosus* fucoidan remained localized as discrete clumps near the cell periphery or within the cells, indicating limited internalization. Cytosolic penetration is a crucial characteristic for anticancer therapeutics because many targets, such as oncoproteins, signaling kinases, and transcription factors, are located deep within the cell [63]. Consequently, the ability to achieve cytosolic penetration provides a significant advantage to the fucoidan derived from *S. filipendula* for its anticancer activities [63].

Overall, the fucoidans from the *S. filipendula* showed effective anticancer activities in MG63 cells, which were higher in the HFSF compared to fucoidan from the *F. vesiculosus*. A different mechanism of action, especially in the case of apoptosis induction and cell penetration, was observed in the study [60].

Multidrug resistance (MDR) remains a critical challenge in cancer treatment. Given the high glycolipid content of seaweed and its potential to reverse this resistance, glycolipid fractions were characterized from *S. filipendula*. Seven glycolipid-positive fractions (GL1-GL7) were evaluated for their inhibition of ABCB1 and ABCC1 efflux using the fluorescent substrates Rhodamine 123 (Rh123) and carboxyfluorescein (CBF), respectively. All fractions except GL1 suppressed ABCB1 and ABCC1 efflux in Lucena-1 cells, and among these, GL2, GL3, and GL4 exhibited the highest activity [38]. The compounds 1-O-(9,12,15-octadecatrienoyl)-3-O-(6-sulfo-α-D-quinovopyranosyl)-glycerol and 1-O-hexadecanoyl-3-O-(6-sulfo-α-D-quinovopyranosyl)-glycerol identified in fraction GL1 do not appear to play a significant role in the inhibition of ABCB1 or ABCC1 mediated efflux. Further, the study investigated the reversal of the drug vincristine (VCR), which is a drug used to treat various types of cancer. The potent ABC-inhibiting fraction GL4 was utilized to evaluate the reversal of VCR resistance in these cells. Notably, co-administration with GL4 significantly sensitized Lucena-1 cells to vincristine, reducing cell viability from 93.6% to 71% at 240 nM and achieving a substantial reduction from 68% to 37.3% at a 960 nM concentration [38].

### 5.4. Antiosteoporotic Activity of S. filipendula

After achieving effective anticancer activities on human osteosarcoma cells, researchers utilized the human embryonic-derived mesenchymal progenitor cells (hES-MP). All components (CFSF, LFSF, MFSF, HFSF, and CFFV) utilized in the anticancer assays were also evaluated for their anti-osteoporotic activity [64]. Early osteogenesis in hES-MPs was assessed via an alkaline phosphatase (ALP) activity assay following a 14-day treatment period; however, no significant or consistent induction of activity was observed. While ALP activity was unchanged at Day 14, Alizarin Red S staining performed on Day 28 revealed significant mineral deposition. Specifically, treatment with 0.1 μg/mL of crude, LMW, and HMW fractions, as well as 0.2 μg/mL of all four *S. filipendula* fucoidans (LMW, MMW, CMW, and HMW), resulted in significantly higher calcium deposition compared to the control. Extensive clinical evidence has established that adequate calcium intake is essential for optimizing bone mineral density and maintaining skeletal integrity [65]. Sirius Red staining was also conducted on Day 28 to evaluate collagen production. The results revealed that while 0.2 μg/mL of MMW fucoidan significantly increased collagen levels compared to the vehicle control, concentrations exceeding this threshold led to a dose-dependent decline in collagen for the MMW, LMW, and HMW fractions. Collagen treatment represents a critical factor in the recovery from osteoporosis, as it contributes to increased bone mineral density and a reduced risk of fractures [66]. Following the observation of significant anti-osteoporotic activity, the cytotoxicity of various fucoidan fractions was evaluated to ensure their safety profile. The toxicity study on hES-MP morphology revealed no significant impact at a dose of 0.2 μg/mL. However, a dose-dependent inhibitory effect was observed at higher concentrations, with the LMW fraction exhibiting the least amount of toxicity. Consistent with the morphological data, assessments of metabolic activity, DNA content, and apoptosis (via Annexin V/PI and Giemsa staining) demonstrated dose-dependent toxicity at elevated levels. Across all assays, the LMW fraction exhibited the lowest levels of toxicity. These results suggest that the biological activity and toxicity of fucoidans vary according to molecular weight [64]. Furthermore, LMW and MMW fractions may effectively promote bone mineralization when used at low concentrations, whereas higher doses may become inhibitory. However, elucidating the underlying mechanism of action will be essential for the development and optimization of these components for therapeutic use. Considering both antiosteoporotic and anticancer activities, researchers suggested future research to study the activity of LMW and MMW, which may be tested on hES-MP (normal cells)/MG63 (cancer cells) co-cultures to determine the optimal molecular weight for dual-purpose therapy [64]. Furthermore, characterizing the signaling pathways involved will be critical to understanding the observed bioactivities.

### 5.5. Immunomodulatory Effects of S. filipendula

An immunosuppressed condition may arise from several factors, including age, lifestyle choices, drug treatments, and malnutrition [67,68]. Such a compromised immune state can leave an individual vulnerable to various infectious and non-infectious diseases [69,70]. Consequently, much like antioxidants, immune-enhancing supplements are gaining significant importance in the modern healthcare scenario. Natural products have shown significant immune enhancement potential as both therapeutic agents and supplements in various studies [71,72].

Building upon previous findings regarding the antioxidant and anticancer properties of heterofucans (SF0.5–SF2) isolated from *S. filipendula*, researchers evaluated the immunomodulatory effects of these fractions in RAW 264.7 murine macrophages [18]. A dose-dependent increase in nitric oxide (NO) production was observed in RAW 264.7 cells following treatment with SF0.5, SF0.7, and SF1. The highest production of NO occurred with SF0.5, reaching levels comparable to the experiment’s positive control. Previous reports in the literature indicate that extracts from other *Sargassum* species (such as *Sargassum horneri* and *Sargassum fusiforme*) significantly stimulate nitric oxide (NO) production [11]. These findings suggest a conserved immune-enhancement pattern that likely extends to *S. filipendula* [11]. In contrast, SF1.5 and SF2 failed to elicit a significant increase in NO production. The sulfate content of these fucans was found to be an insignificant factor in stimulating NO release, as no correlation was observed between NO production and the degree of sulfation. Although the density of sulfate groups influences activity, their spatial distribution across the polysaccharide backbone is a more significant factor in determining whether the compound exhibits high or low biological efficacy. Notably, heterofucans with higher xylose and glucuronic acid content exhibited greater NO stimulatory action. This was confirmed by a strong positive Pearson correlation between NO levels and xylose (r = 0.98) or glucuronic acid (r = 0.92).

Treatment with SF0.5 and SF0.7 significantly increased the levels of TNF-α and IL-6 in RAW 264.7 cells, as measured by ELISA. SF0.5 showed the highest increase in the levels of TNF-α and IL-6, which was better than the reported activity of fucans from the other species, such as *F. vesiculosus* and *A. nodosum* [18]. Furthermore, the immunomodulatory activities demonstrated by various *Sargassum* species in both in vitro and in vivo models provide a robust foundation for their therapeutic potential [11,73]. These findings suggest that active fractions of *S. filipendula* warrant further investigation through more rigorous animal studies to evaluate their efficacy and safety. However, SF0.7, SF1.5, and SF2 failed to elicit a significant increase in the levels of TNF-α and IL-6. Similar to NO production, strong positive Pearson correlations were observed between TNF-α or IL-6 levels and the xylose and glucuronic acid content of the studied heterofucans.

Importantly, immunomodulation is a complex phenomenon involving multiple cells, tissues, and organs associated with the immune system, such as the thymus and spleen [74]. This inherent complexity limits the findings of cell line-based studies; therefore, the validation of the immune enhancement activity of *S. filipendula* is highly suggested to be evaluated using established immunosuppression models in animals. A prominent example is the cyclophosphamide-induced immunosuppression model in mice, which systematically exerts toxic effects on the spleen and thymus to produce a controlled immunosuppressed condition in vivo [74]. By utilizing such a model, researchers can more accurately observe the holistic response of the immune system to *S. filipendula* treatment, accounting for the intricate interactions between different lymphatic organs and circulating immune cells.

### 5.6. Antimicrobial Activity

Infectious diseases remain a primary public health concern due to their rapid transmission rates, their profound impact on global economic stability, the escalating threat of antimicrobial resistance, and the continuous emergence of novel pathogens [75,76,77]. A growing body of research has demonstrated that natural resources, ranging from plant-derived secondary metabolites to marine compounds, possess potent antimicrobial properties. These bioactive agents have shown significant efficacy in inhibiting a broad spectrum of pathogens, including bacteria [78] and fungi [79]. The antimicrobial activities of *S. filipendula* across all these types of organisms have been studied in various studies.

#### 5.6.1. In Vitro Anti-Bacterial Activity

In a screening study, the in vitro antibacterial activity of the ethyl acetate fraction, derived from a methanol extract of *S. filipendula*, was evaluated against *Staphylococcus aureus*, *Bacillus subtilis*, *Streptococcus agalactiae*, *Escherichia coli*, *Pseudomonas aeruginosa*, *Klebsiella pneumoniae*, and *Shigella flexneri*, along with its antifungal activity [80]. The minimum inhibitory concentration (MIC) and minimum bactericidal concentration (MBC) values were determined for the selected bacterial species, which revealed that the fraction exhibited antibacterial activity specifically against two Gram-positive bacteria: *S. aureus* and *B. subtilis* (Table 2) [80]. The selectivity of the fraction used towards *S. aureus* and *B. subtilis* (Gram-positive bacteria) suggests a specialized mechanism of action targeting strain-specific pathways rather than conserved bacterial components [80]. Elucidating these molecular targets through future mechanistic studies will be essential to optimize *S. filipendula* as a potent, targeted antibacterial agent.

Later, the inhibition of growth in two bacterial species, *Staphylococcus epidermidis* and *Klebsiella pneumoniae*, was studied. The tested heterofucans were not able to suppress the growth of these bacterial species in the study [18]. However, biofilm formation by *Staphylococcus epidermidis* was significantly (~50%) inhibited by SF0.5 [18]. These results raise an interesting question: why is the activity confined to *Staphylococcus epidermidis*, and why does it not affect biofilm formation in *Klebsiella pneumoniae*? *Klebsiella* is a Gram-negative bacterium, whereas *Staphylococcus epidermidis* is a Gram-positive bacterium. The structural differences in the bacterial cell walls may be the reason for this selective activity [81]. Further studies are required on other Gram-positive bacteria to confirm whether this effect is specific to Gram-positive bacteria.

The oven-dried *S. filipendula* samples were extracted using various solvents, including ethanol, methanol, and diethyl ether, to investigate their antibacterial properties [36]. Eleven bacterial isolates, collected from wastewater, were used for the study. Antibacterial activity was determined by measuring the zone of inhibition around each well, which indicated the antimicrobial potency of the extracts. The diethyl ether extract was found to be the most effective, exhibiting activity against 10 out of the 11 bacterial isolates obtained from wastewater. Based on the highest zones of inhibition, three specific isolates were identified via 16S rRNA sequencing as *Bacillus amyloliquefaciens*, *Chryseobacterium cucumeris*, and *Bacillus cereus* [36].

The antibacterial activity of extracts from four different seaweeds, including *S. filipendula*, was evaluated against three human pathogenic bacterial species: *Escherichia coli*, *Salmonella typhimurium*, and *Staphylococcus aureus*. Bacterial growth was evaluated using the pour plate technique, and the percentage inhibition was determined according to the formula Percentage inhibition = [(Cc − Cs)/Cc] × 100, where Cc represents the colony count of the control and Cs represents the colony count of the treated sample. Dose-dependent antibacterial activity was observed against all three bacterial species evaluated in this study. Among the four seaweed extracts, the *S. filipendula* extract exhibited the maximum inhibitory activity, reaching 85.7% against *E. coli* at a concentration of 100 μg/mL. Furthermore, the *S. filipendula* extract showed 82.5% inhibition against *S. aureus* and 65% against *S. typhimurium* at the same concentration [37]. Various antibacterial assays revealed that *S. filipendula* exhibits potent activity against the majority of tested strains (*B. subtilis*, *E. coli*, *S. aureus*, *S. typhimurium*, *B. amyloliquefaciens*, *Chryseobacterium cucumeris*, and *B. cereus*). However, certain Gram-negative bacteria (such as *Klebsiella pneumoniae*) remained unaffected by the *S. filipendula* components [18]. This selectivity highlights the need to investigate the underlying molecular mechanisms, which will be essential for exploring the specific mode of action and further optimizing *S. filipendula* for pharmaceutical development.

#### 5.6.2. In Vitro Antifungal Activity

The in vitro antifungal activity of the ethyl acetate fraction, derived from a methanol extract of *S. filipendula*, was evaluated against *Candida albicans*, *Saccharomyces cerevisiae*, *Aspergillus niger*, and *Trichophyton mentagrophytes*, along with its antibacterial activity [80]. Both the MIC and minimum fungicidal concentration (MFC) were determined for the selected fungal species, which revealed that the fraction exhibited antifungal activity specifically against *T. mentagrophytes*, which is considered a highly pathogenic fungal species (Table 2). Similar to its antibacterial profile, the selective antifungal activity of *S. filipendula* suggests a specialized mechanism of action. This selectivity indicates that *S. filipendula* may target specific pathways unique to certain fungi, rather than acting on conserved components common across diverse fungal species. Such findings highlight the necessity for future mechanistic studies to elucidate these specific targets. Characterizing the molecular mechanism of action will be critical for the strategic optimization of *S. filipendula* as a potent and targeted antifungal agent.

### 5.7. In Vitro Antiprotozoal Activity

Inspired by the immunomodulatory properties of heterofucans (SF0.5–SF2) isolated from *S. filipendula*, researchers evaluated their antiprotozoal activity against trophozoites of *Trichomonas vaginalis* [18]. Treatment with SF0.7, SF1, and SF1.5 exhibited significant anti-*Trichomonas vaginalis* activity, which was highest in the case of SF0.7 [18]. The sugar-to-sulfate ratio was positively correlated with anti-protozoal activities, suggesting that the sulfation of heterofucans plays a key role in their bioactivity, similar to their antioxidant properties [18]. The antiprotozoal activity of SF may also extend to other important protozoal parasites, such as *Leishmania donovani* [82]. Furthermore, detailed mechanistic studies may be helpful to understand the molecular targets and optimize this activity in the near future.

### 5.8. In Vitro Antiviral Activity

Aqueous and methanolic extracts of *S. filipendula* were prepared following 10 days of treatment under three distinct conditions: exposure to UVA + PAR, UVB + PAR, and PAR only (control). These extracts were evaluated for antiviral activity against the HIV-1 reverse transcriptase enzyme. High inhibitory activities against RT (100% at 50 μg/mL) were exhibited by the aqueous extracts prepared across all conditions. Regarding the methanolic extracts, the control group exhibited the highest inhibition, followed by the UVA and UVB groups. This suggests that UV exposure does not stimulate antiviral activity in these extracts [80].

The antiviral activity of extracts prepared from four different seaweeds, including *S. filipendula*, was evaluated in conjunction with their antibacterial activity. These extracts were prepared using an equal solvent mixture of methanol and hexane. Prior to assessing antiviral activity, the cytotoxicity of the extracts was evaluated on Vero and MA104 cell lines. Among the four seaweeds tested, *S. filipendula* exhibited the lowest toxicity toward both cell lines; specifically, it showed the highest cytotoxic concentration (CC_50_). The antiviral activity was evaluated on both cell lines against rotavirus and coxsackievirus B3 (CVB3). The half-maximal effective concentration (EC_50_), defined as the extract concentration that protects 50% of infected cells, was calculated. Finally, the therapeutic index (TI) of each seaweed extract was determined by calculating the ratio of CC_50_ to EC_50_ to quantify antiviral efficacy and safety. The TI values for the *S. filipendula* extracts were 1.4 for rotavirus and 2.4 for CVB3 [37]. The relatively low antiviral activity of the *S. filipendula* extract necessitates further optimization for pharmaceutical development. This may be achieved by isolating the specific bioactive components previously identified through GC-MS analysis. Furthermore, network pharmacology coupled with molecular docking studies against viral protease or capsid proteins, followed by experimental validation, could prove instrumental in this process.

**Table 2:** Pharmacological activities reported in *S. filipendula*.

Sr. No.	Activity	Type of Extract/Fraction	Methods	Results	References
1	Antiaging	Purified fucoidan, crude fucoidan, and crude polyphenolic extracts of SF	Matrix Metalloproteinases (MMPs) Inhibition (collagenase inhibition assay)	The IC_50_ values were 0.17 ± 0.033, 9.97 ± 0.16, 1.61 ± 0.00, and 0.36 ± 0.01 mg/mL for EGCG, pure fucoidan, crude fucoidan, and crude polyphenol extracts, respectively.	[16]
			Elastase inhibition assay	The IC_50_ values were 0.4 ± 0.01, 0.04 ± 0.02, and 0.04 ± 0.01 mg/mL for EGCG, crude fucoidan, and crude polyphenol extracts, respectively.	
2	Antioxidant activity	Sulfated polysaccharides	Reducing Power	110% activity of ascorbic acid.	[17]
		Heterofucans (SF0.5, SF0.7, SF1, SF1.5, and SF2) isolated from SF	TAC assay	TAC valued were ~46, 77.3, 90.7, ~59, and 9.6 ascorbic acid equivalents for SF0.5, SF0.7, SF1, SF1.5, and SF2, respectively.	[19]
			Scavenging (%) OH˙	OH˙ scavenging percents were 26.2 ± 1.8, 26.7 ± 1.8, and 12.7 ± 4.8 for SF0.7, SF1, SF1.5, respectively at 0.5 mg/mL.	
			Scavenging (%) O_2_^−^	O_2_^−^ scavenging percents were 19.3 ± 0.7 and 12.2 ± 1.2 for SF0.7 and SF2, respectively at 0.5 mg/mL.	
			Chelating Effect on Ferrous Ions	54.8% ferrous chelation at 2.0 mg/mL for SF2.	
			Reducing Power	>90.5% at 0.5 mg/mL for SF1.	
		Fucoxanthin isolated from SF	DPPH assay	IC_50_ = 1.4174 ± 0.0126 μg/mL	[33]
		Crude fucoidan extract, sulfonated fucoidan and standard fucoidan in DDW	DPPH assays	Scavenging Effect were 4.3, 4.6, and 12.2%, for Crude fucoidan extract, sulfonated fucoidan and standard fucoidan, respectively.	[41]
		Crude fucoidan extract, sulfonated fucoidan and standard fucoidan in DMSO		Scavenging Effect were 8.9%, 10.9, and 75.1%, for Crude fucoidan extract, sulfonated fucoidan and standard fucoidan, respectively.	
		Fucoidan isolated from SF	DPPH assays	IC_50_: 85.46 ± 4.17 μg/mL	[44]
		Methanol extract of SF from control, UVA and UVB exposer for 10 days	DPPH	EC_50_ values were 0.76 ± 0.02, 0.89 ± 0.02, and 1.33 ± 0.07 μg/mL for control, UVA and UVB groups, respectively	[29]
			ABTS	EC_50_ values were 0.62 ± 0.03, 0.73 ± 0.03, and 0.83 ± 0.04 μg/mL for control, UVA and UVB groups, respectively	
			FRAP	EC_50_ values were 0.61 ± 0.07, 0.68 ± 0.05, and 1.09 ± 0.09 μg/mL for control, UVA and UVB groups, respectively	
			Ferrous ion chelation	Chelation values were 10.0 ± 3, 8.7 ± 2.6, and 8.6 ± 4.2 mg GAE/g for control, UVA and UVB groups, respectively	
		Purified fucoidan, crude fucoidan, and crude polyphenolic extracts of SF	DDPH scavenging activities	EC_50_ values for pure fucoidan, crude fucoidan, and crude polyphenol extracts were 8.19 ± 0.033, 2.90 ± 0.01, and 2.83 ± 0.01, and 0.571 ± 0.050 mg/mL, respectively.	[16]
			Lipid peroxidation of methyl Linoleate MDA	Values for crude fucoidan, and crude polyphenol extracts were 0.28 ± 0.01 and 0.50 ± 0.16, mmol MDA/kg MeLo	
			Lipid peroxidation of methyl Linoleate CDH	Values for crude fucoidan, and crude polyphenol extracts were 19.97 ± 2.97 and 57.46 ± 2.36 mg/mL mmol CDH/kg MeLo	
			Superoxide radical,	EC_50_ values for crude fucoidan, and crude polyphenol extracts were 1.49 and 2.75 mg/mL	
			ABTS radical,	EC_50_ values for pure fucoidan, crude fucoidan, and crude polyphenol extracts were 10.1 ± 0.26, 1.86 ± 0.026, and 2.90 ± 0.02 mg/mL.	
		SF flours were prepared via oven-drying (SD) and freeze-drying (SL).	DPPH	Value for SD and SL were 470.58 ± 6.29 and 578.08 ± 6.29 μmol TE/g, respectively.	[20]
			ABTS	Value for SD and SL were 831.66 ± 1.67 and 1001.66 ± 1.67~μmol TE/g, respectively.	
			FRAP	Value for SD and SL were 390.81 ± 2.80 and 435.63 ± 2.80 μmol TE/g, respectively.	
3	Anticancer	Heterofucan	MTT assay of HeLa cells	61.1% inhibition of cell proliferation at 0.1 mg/mL.	[17]
			MTT assay of HeLa cells	72.5% inhibition of cell proliferation (at 0.1 to 2.0 mg/mL)	[54]
			Annexin V/PI double staining.	Apoptosis ↑	
			WB	Phosphorylation of GSK-3β ↓ AIF ↑ (in cytosol)	
		Heterofucans (SF0.5, SF0.7, SF1, Sf1.5, and SF2) isolated from SF	MTT assay of HeLa, PC3, and HepG2 cell lines	Inhibition of all cell line was observed by heterofucans (only SF0.7 does inhibit HeLa cells)	[19]
		Fucoxanthin isolated from SF	TUNEL assay for HeLa cells	Apoptosis ↑ (100% at 50–100 ppm)	[33]
		Fucoidan isolated from SF	MG63 cells Presto Blue assay	Metabolic activity ↓	[60]
			DNA Content Assay	DNA Content ↓	
			Giemsa staining stained MG63	Altered morphology and cell death ↑	
			TEM analysis	Blebbing in the cell membrane, extensive ER-like swelling, signs of autophagosome formation, chromatin condensation and marginalisation. mitochondria were again dense and seemed to have swollen cristae structures	
			JC-1 staining	Mitochondrial membrane depolarization	
			Immunostaining fucoidans	Extensive cytosolic penetration	
		Glycolipid fractions (GL1-7) of SF extract	ABC-mediated efflux assay in Lucena-1 cells	Suppression of ABC transporter activity	[38]
		Glycolipid fraction GL4 of SF extract co administered with vincristine	MTT assay ofLucena-1 cells	Cell viability ↓	[38]
4	Immunomodulatory	Heterofucans (SF0.5, SF0.7, SF1, Sf1.5, and SF2) isolated from SF	RAW 264.7 cells NO was analyzed through quantification of nitrite production by the Griess reaction	NO ↑ (by SF0.5, SF0.7, and SF1.)	[18]
			ELISA	TNF-α ↑ and IL-6 ↑ (by SF0.5 and SF0.7)	
5	Antibacterial	Ethyl ether fraction of methanol extract	MIC and MBC against *Staphylococcus aureus*, *Bacillus subtilis*, *Streptococcus agalactiae*, *Escherichia coli*, *Pseudomonas aeruginosa*, *Klebsiella pneumoniae*, and *Shigella flexneri*	MIC of 1.56 mg/mL and an MBC > 12.5 mg/mL against *S. aureus*, while for *B. subtilis*, the MIC was 3.13 mg/mL with an MBC > 12.5 mg/mL	[80]
		Heterofucans (SF0.5, SF0.7, SF1, Sf1.5, and SF2) isolated from SF	Biofilm formation by *Staphylococcus epidermidis*	Biofilm formation ↓ (by SF0.5)	[18]
		Diethyl ether extract of SF	Zones of inhibition of bacterial isolotes from the waste water (includes, *Bacillus amyloliquefaciens*, *Chryseobacterium cucumeris*, and *Bacillus cereus*)	Bacterial growth ↓	[36]
		SF extracted through methanol/hexane solvent	antibacterial activity *Escherichia coli*, *Salmonella typhimurium*, and *Staphylococcus aureus*	85.7%, 82.5%, and 65% against *E. coli*, *S. aureus* and *S. typhimurium,* respectively.	[37]
6	Antiprotozoal	Heterofucans (SF0.5, SF0.7, SF1, Sf1.5, and SF2) isolated from SF	Anti-*Trichomonas vaginalis* assay (counting trophozoites through fluorescence spectrophotometer)	Trophozoites ↓ (by SF0.7, SF1, and SF1.5)	[18]
7	Antiviral	Aqueous and ethanol extract of SF control, UVA and UVB exposer for 10 days	Inhibition of reverse transcriptase of HIV-1	IC_50_ values were 264.77 ± 15.07, 589.70 ± 56.14, and 417.40 ± 6.50 μg/mL for control, UVA, UVB groups for methanol extracts and 100% inhibition at 50 by aqueous extracts.	[29]
		SF extracted through methanol/hexane solvent	Rotavirus and CVB3 infected Vero and MA104 cell lines	TI values were 1.4 and 2.4 for Rotavirus and CVB3. EC_50_ values were 924 and 353.3 μg/mL for Rotavirus and Coxsackievirus B3 (CVB3), respectively.	[37]
8	Antifungal	Ethyl ether fraction of methanol extract	MIC and MFC against *Candida albicans*, *Saccharomyces cerevisiae*, *Aspergillus niger*, and *Trichophyton mentagrophytes*	*Trichophyton mentagrophytes* ↓	[80]
9	Antiosteoporotic activity	CFSF, LFSF, MFSF, HFSF (0.1–1 μg/mL)	Alizarin Red S staining of hES-MP cells performed on Day 28	Mineral deposition ↑ (at 0.2 μg/mL)	[64]
			Sirius Red staining for hES-MP cells was also conducted on Day 28	Collagen levels ↑ (0.2 μg/mL of MFSF)	

AIF: apoptosis-inducing factor; CVB3: Coxsackievirus B3; GSK-3β: glycogen synthase kinase-3 beta; hES-MP: human embryonic-derived mesenchymal progenitor cells; MBC: minimum bactericidal concentration; MFC: minimum fungicidal concentration; MIC: minimum inhibitory concentration; MTT: 3-(4,5-Dimethylthiazol-2-yl)-2,5-diphenyltetrazolium bromide; PI: propidium iodide; SF: *Sargassum filipendula*; TE: trolox equivalent; TI: therapeutic index; TUNEL: terminal transferase deoxyuridine triphosphate nick deoxynucleotide end labeling; ↑: up-regulation; ↓: down-regulation.

## 6. Toxicity and Possible Contamination

Studies have demonstrated that *S. filipendula* possesses a rich nutrient profile and an array of potential pharmacological properties. While toxicity studies specifically focused on *S. filipendula* are limited, acute toxicity assessments of *Sargassum* spp. extracts in a mouse model reported no mortality throughout the experimental period, even at the highest dose of 2000 mg/kg BW [30]. Nevertheless, the genus is prone to bioaccumulating pollutants, especially heavy metals (As, Cd, Pb, and Hg) from contaminated marine environments, posing a potential health risk to humans upon ingestion [83]. *S. filipendula* can readily accumulate toxic metals, including lead and cadmium, from the surrounding environment [84,85]. This property makes *S. filipendula* an environmentally friendly and cost-effective alternative for treating aqueous solutions containing toxic metals. However, it may also pose a risk of metal toxicity for human consumption, especially if collected from environments polluted with heavy metals [83,84]. Toxicological studies have reported symptoms of metal toxicity in rats following the ingestion of *Sargassum fusiforme*, and several food safety authorities have established guidelines and advisories concerning its dietary intake [86]. The risk of heavy metal toxicity is a common challenge across various seaweed taxa; thus, *S. filipendula* requires precautionary measures when harvested for nutritional or medicinal purposes. Potential solutions to mitigate toxicity include selective breeding, controlled farming, and advanced post-harvest processing [83]. Implementing routine testing, mandatory labeling, and updated regulatory monitoring will safeguard the benefits of seaweed while minimizing toxicological risks [83].

## 7. Conclusions and Future Directions

The potent antioxidant activity of *S. filipendula* extracts is primarily attributed to fucoidans and phytochemicals, such as phlorotannins and fucoxanthin. This activity correlates closely with fucoidan content; furthermore, the sulfation degree of fucoidan appears to enhance this effect, as sulfated fucoidans exhibit significantly increased antioxidant capacity. Similarly, fucoxanthin isolated from *S. filipendula* demonstrated antioxidant activity comparable to that of β-carotene. However, the enzymatic antioxidant capacity of *S. filipendula* remains unexplored. The evaluation of the impact of *S. filipendula* on antioxidant enzyme expression and activity using diverse cell lines and in vivo models is necessary to fully characterize its potential as a therapeutic agent or nutritional supplement.

It can also be concluded that abiotic stressors, such as UV exposure, may suppress the TPC of *S. filipendula*, consequently reducing its pharmacological potential, including its antioxidant activity observed with various methods [29].

Regarding immunomodulatory activity, the xylose and glucuronic acid present in the fraction were positively correlated with NO production, as well as TNF-α and IL-6 levels, suggesting that these compounds play a significant role in immunomodulation. Immunomodulation and antioxidant capacity are promising primary activities, likely acting as fundamental contributors to the broader pharmacological profile of *S. filipendula* [87]. However, the immune-enhancing potential of *S. filipendula* has yet to be validated in animal studies, which are essential for confirming this effect. Consequently, it is recommended that *S. filipendula* be evaluated in mice models of immunosuppression induced by agents such as methotrexate, 5-fluorouracil, or cyclophosphamide, as well as in other disease models associated with immune deficiency [71,72,88].

Anti-inflammatory activity plays a critical role in combating several chronic diseases and is essential for maintaining physiological homeostasis [89]. While numerous *Sargassum* species have demonstrated anti-inflammatory activities in the literature, no direct studies have specifically evaluated the anti-inflammatory potential of *S. filipendula* [90]. This represents a significant research gap, as such activity may be a key contributing factor to the diverse pharmacological properties of the species. Conversely, contrasting immunomodulatory activities, such as enhanced NO production and increased cytokine expression, have been reported for heterofucans isolated from *S. filipendula* [18]. Therefore, the anti-inflammatory potential of various *S. filipendula* components should be rigorously evaluated in animal models of inflammation in future studies. The antimicrobial activity of *S. filipendula* components against a wide range of pathogens, including fungi, and various bacterial species, renders it highly significant and promising for further development. The antibacterial activity of *S. filipendula* was effective against most of the bacterial species tested, although it was inactive against one Gram-negative species [18]. Similarly, the antiviral effectiveness varied among the tested viruses [88], and antifungal activity was not observed against the two opportunistic pathogenic species included in the experiments [80]. These results suggest that SF may act upon pathogen-specific targets. Therefore, understanding the molecular mechanisms and specific targets will be essential for the further optimization and development of its antimicrobial properties. Considering the broad-spectrum efficacy of *S. filipendula* demonstrated in various studies, it is recommended that future research expand to include a wider array of pathogenic bacteria and fungal species. Furthermore, investigating these additional microbial pathogens is essential for identifying potential cross-kingdom mechanisms of action, which could position SF as a versatile template for the development of multi-target antimicrobial therapies.

Notably, studies have revealed that supplementation with *S. filipendula* has a positive impact on the gut microbiome of shrimp [91,92]. However, while brown seaweeds can contribute pharmacological effects by modulating the mammalian gut microbiota, the specific bioactivity of *S. filipendula* in relation to these microbial shifts has not yet been investigated in a mammalian model [93,94,95]. Further studies using animal models to evaluate the impact of *S. filipendula* supplementation on the gut microbiome are suggested to comprehensively understand the mechanisms supporting its pharmacological activities.

While studies have identified several phytochemicals from *S. filipendula* extracts that support its pharmacological profile, many constituents remain uncharacterized due to their low concentrations and poor resolution during GC-MS analysis. Future investigations should identify these minor components to determine their specific or synergistic roles in the extract’s overall bioactivity.

Importantly, *S. filipendula* readily accumulates toxic metals from its environment, posing a significant risk to human health, especially when harvested from polluted regions. Given that toxicological studies have reported metal-induced toxicity in rats following the ingestion of *Sargassum fusiforme*, precautionary measures are essential when utilizing *S. filipendula* for nutritional or medicinal purposes. Furthermore, strategies such as advanced post-harvest processing, routine testing, and updated regulatory monitoring are suggested to safeguard the seaweed’s therapeutic benefits while minimizing toxicological risks.

The practical application of *S. filipendula* as a functional food is well-supported, given its impressive nutritional profile and diverse pharmacological activities. Furthermore, recent studies have successfully demonstrated its utility in enhancing the nutritional value of rice flour and various other food products. However, data regarding scalability and standardized processing methods remain scarce, representing a significant barrier to the large-scale industrial application of *S. filipendula*. Furthermore, limited toxicological assessments and complex regulatory frameworks are critical hurdles that must be addressed to facilitate commercial development [83]. Most importantly, the paucity of in vivo studies and the lack of clinical validation for its pharmacological activities remain the primary constraints restricting the use of *S. filipendula* as a therapeutic agent or dietary supplement at any scale.

In conclusion, *S. filipendula* possesses diverse pharmacological properties that necessitate further validation through comprehensive animal studies to establish its therapeutic efficacy. Future research should prioritize the elucidation of the underlying molecular mechanisms alongside the determination of optimized dosage regimens and rigorous safety profiles. By establishing a clear relationship between the bioactivity and its metabolic pathways in vivo, these studies may provide a robust scientific foundation for the eventual transition of *S. filipendula* to clinical evaluation as a potential therapeutic agent.

## Data Availability

Data are contained within the article.
